# Soya foods and breast cancer risk: a prospective study in Hiroshima and Nagasaki, Japan

**DOI:** 10.1038/sj.bjc.6690837

**Published:** 1999-12

**Authors:** T J Key, G B Sharp, P N Appleby, V Beral, M T Goodman, M Soda, K Mabuchi

**Affiliations:** Imperial Cancer Research Fund Cancer Epidemiology Unit, Gibson Building, Radcliffe Infirmary, Oxford, OX2 6HE, UK; Department of Epidemiology, Radiation Effects Research Foundation, 5-2 Hijiyama Park, Minami-ku, Hiroshima, 732-0815, Japan; Cancer Research Center of Hawaii, 1236 Lauhala Street, Honolulu, HI, 96813, USA; Department of Epidemiology, Radiation Effects Research Foundation, Nagasaki 850, Japan

**Keywords:** breast cancer, soya, phyto-oestrogens, atomic bomb survivors, Japan

## Abstract

The association between soya foods and breast cancer risk was investigated in a prospective study of 34 759 women in Hiroshima and Nagasaki, Japan. Women completed dietary questionnaires in 1969–1970 and/or in 1979–1981 and were followed for incident breast cancer until 1993. The analysis involved 427 cases of primary breast cancer in 488 989 person-years of observation. The risk for breast cancer was not significantly associated with consumption of soya foods: for tofu, relative risks adjusted for attained age, calendar period, city, age at time of bombings and radiation dose to the breast were 0.99 (95% CI 0.80–1.24) for consumption two to four times per week and 1.07 (0.78–1.47) for consumption five or more times per week, relative to consumption once a week or less; for miso soup, relative risks were 1.03 (0.81–1.31) for consumption two to four times per week and 0.87 (0.68–1.12) for consumption five or more times per week, relative to consumption once a week or less. These results were not materially altered by further adjustments for reproductive variables and were similar in women diagnosed before age 50 and at ages 50 and above. Among 17 other foods and drinks examined only dried fish (decrease in relative risk with increasing consumption) and pickled vegetables (higher relative risk with higher consumption) were significantly related to breast cancer risk; these associations were not prior hypotheses and, because of the large number of comparisons made, they may be due to chance. © 1999 Cancer Research Campaign


*British Journal of Cancer* (1999) **81**(7), 1248–1256
© 1999 Cancer Research Campaign
Article no. bjoc.1999.0837

### Soya foods and breast cancer risk: a prospective study in Hiroshima and Nagasaki, Japan

**TJ Key[1], GB Sharp[2], PN Appleby[1], V Beral[1], MT Goodman[3], M Soda[4] and K Mabuchi[2]**

[1]Imperial Cancer Research Fund Cancer Epidemiology Unit, Gibson Building, Radcliffe Infirmary, Oxford, OX2 6HE, UK; [2]Department of Epidemiology, Radiation Effects Research Foundation, 5-2 Hijiyama Park, Minami-ku Hiroshima, 732-0815 Japan; [3]Cancer Research Center of Hawaii, 1236 Lauhala Street, Honolulu, HI 96813, USA; [4]Department of Epidemiology, Radiation Effects Research Foundation, Nagasaki 850, Japan

**Summary** The association between soya foods and breast cancer risk was investigated in a prospective study of 34 759 women in Hiroshima and Nagasaki, Japan. Women completed dietary questionnaires in 1969–1970 and/or in 1979–1981 and were followed for incident breast cancer until 1993. The analysis involved 427 cases of primary breast cancer in 488 989 person-years of observation. The risk for breast cancer was not significantly associated with consumption of soya foods: for tofu, relative risks adjusted for attained age, calendar period, city, age at time of bombings and radiation dose to the breast were 0.99 (95% CI 0.80–1.24) for consumption two to four times per week and 1.07 (0.78–1.47) for consumption five or more times per week, relative to consumption once a week or less; for miso soup, relative risks were 1.03 (0.81–1.31) for consumption two to four times per week and 0.87 (0.68–1.12) for consumption five or more times per week, relative to consumption once a week or less. These results were not materially altered by further adjustments for reproductive variables and were similar in women diagnosed before age 50 and at ages 50 and above. Among 17 other foods and drinks examined only dried fish (decrease in relative risk with increasing consumption) and pickled vegetables (higher relative risk with higher consumption) were significantly related to breast cancer risk; these associations were not prior hypotheses and, because of the large number of comparisons made, they may be due to chance. © 1999 Cancer Research Campaign

**Keywords**: breast cancer; soya; phyto-oestrogens; atomic bomb survivors; Japan

The risk for breast cancer is increased by exposure to high levels of endogenous and exogenous oestrogens (Pike et al, 1993; Collaborative Group on Hormonal Factors in Breast Cancer, 1997; Thomas et al, 1997). Soya foods are rich in precursors of the isoflavones daidzein and genistein, which are heterocyclic phenols similar in structure to oestrogens, and it has been hypothesized that a high dietary intake of soya foods might reduce breast cancer risk by interfering with the action of endogenous oestradiol (Setchell et al, 1984). The results of studies of the effects of dietary isoflavones on oestrogen levels and ovulatory function have varied, but some studies have suggested that high intakes in premenopausal women may reduce serum oestradiol concentrations, suppress the mid-cycle surge of gonadotrophins, and perhaps increase the length of the menstrual cycle (Cassidy et al, 1994, 1995; Lu et al, 1996; Nagata et al, 1997*a*).

Several studies have reported on the association of soya foods with breast cancer risk, but the results have been inconsistent (Nomura et al, 1978; Hirohata et al, 1985; Hirayama, 1990; Lee et al, 1991, 1992; Hirose et al, 1995; Yuan et al, 1995; Greenstein et al, 1996; Wu et al, 1996; Witte et al, 1997). We report here the association between soya foods and breast cancer risk among 34 759 women in the Life Span Study cohort in Hiroshima and Nagasaki, Japan. A previous paper reported on the association of breast cancer risk with reproductive factors and radiation exposure in 22 200 of these women who completed a questionnaire in 1979–1981 (Goodman et al, 1997); the current analysis is an extension of that work that uses the risk factors identified in the previous analysis as covariates in the examination of dietary factors and includes women who completed the earlier 1969–1970 questionnaire in addition to women who completed the 1979–1981 questionnaire.

*Received 7 April 1999*
*Revised 13 May 1999*
*Accepted 18 May 1999*

*Correspondence to:* TJ Key

### MATERIALS AND METHODS

### Subjects

The Radiation Effects Research Foundation's Life Span Study is a cohort of approximately 50 000 men and 70 000 women. Of these, 93 741 were both present in Hiroshima or Nagasaki at the time of the bombings and city residents on 1 October 1950, the time of the first post-war national census (Preston et al, 1987). The cohort also includes 26 580 residents not present in either city at the time of the bombings who were identified in special censuses conducted between 1950 and 1953 (Preston et al, 1987). The data used in the current analysis are from two mail surveys conducted among the Life Span Study cohort to collect further information on characteristics of the subjects including lifestyle, reproductive factors and dietary habits.

The first mail survey of women in the Life Span Study (Survey 1) was sent to the 39 000 women alive on 1 September 1969, with mailing between 1969 and 1970. A total of 20 832 (53.4%) women responded to this survey. The second mail survey of women in the Life Span Study (Survey 2) was sent to the 34 421 women alive in September 1979 in Hiroshima and in July 1979 in Nagasaki. Three mailings were made to maximize the response. The last completed questionnaires were received in February 1981. A total of 24 995 women (72.6%) responded to this survey. Women who were absent from the cities at the time of the bombings were included in Survey 1 but excluded from Survey 2. Altogether, 34 759 women completed one or both questionnaires: of these, 9 765 completed the Survey 1 questionnaire only, 13 927 completed the Survey 2 questionnaire only, and 11 067 completed both questionnaires.

### Questionnaires on lifestyle, reproductive factors and diet

The mail survey questionnaires sent out in 1969–1970 (Survey 1) and in 1979–1980 (Survey 2) were very similar; indeed most questions were identical in the two questionnaires. The questionnaires covered reproductive history, weight and height, and other possible risk factors for breast cancer including use of exogenous hormones, smoking and alcohol consumption.

Both the mail survey questionnaires included a section on diet. The list of foods was similar on the two questionnaires. The analyses described here report the relative risk for breast cancer in relation to consumption of nineteen foods and drinks: tofu (soya bean curd); miso soup (miso is fermented soyabean paste); ham, sausage etc.; other meat (including chicken in Survey 2 only); fish (sashimi (raw fish), boiled, fried etc., excluding broiled or dried fish); dried fish; milk; butter and cheese (excluding margarine); eggs; rice; bread; Western-style confectionery; fruit; green or yellow vegetables (pumpkin, carrot, spinach, etc.; included in the Survey 2 questionnaire only); sea vegetables; pickled vegetables (and salted fish gut); coffee; black tea; and green tea.

For rice and bread, the frequencies of consumption were: once or less per day; twice per day; three times or more per day. For green tea, the frequencies were: once or less per day; two to four times a day; five times or more per day. For the other foods, the frequencies were once or less per week; two to four times per week; five or more times per week. In the Survey 2 questionnaire each food and drink had an additional category of never consumed; for compatibility with the Survey 1 data, this category was combined with the next lowest intake category.

### Radiation dose estimates

Radiation dose estimates were based on the Dosimetry System 1986 (DS86), which is the result of refinements in radiation doses made by several working groups in Japan and the USA in the 1980s (Roesch, 1987). The DS86 system breast dose from gamma rays and neutrons is based on physical calculations of yield with individual data on shielding by buildings, terrain and body tissue. We assumed a quality factor (relative biological effectiveness) of 10 for neutrons (i.e. breast dose = gamma dose + 10 × neutron dose) to allow for their differential effectiveness. The actual relative biological effectiveness may vary by dose, but precise values for the breast are unknown.

### Follow-up

Follow-up for cancer incidence was by linkage with the population-based cancer registries in Hiroshima and Nagasaki (Mabuchi et al, 1994). Follow-up for mortality was by linkage with the Japanese family registration system. This provides virtually complete ascertainment of death and cause of death as recorded on the death certificate for all subjects still living in Japan.

For women who responded to Survey 1, the start of follow-up was taken as the month and year in which the completed questionnaire was received. For women who did not respond to Survey 1 but who did respond to Survey 2, the start of follow-up was taken as February 1981 for Hiroshima and November 1981 for Nagasaki because the exact dates of receipt of the completed questionnaires were not recorded and these were the latest months in which questionnaires were received. The end of follow-up was taken as the date of diagnosis of breast cancer, date of diagnosis of any other malignant neoplasm, date of death, or 31 December 1993, whichever occurred first. Women who were registered with cancer before the start of follow-up were excluded.

### Adjustment for migration

For subjects whose follow-up ends in the diagnosis of cancer, it is known that they have not migrated out of the catchment areas of the cancer registries. For other subjects, whose follow-up ends in death or on 31 December 1993, it is unknown whether they are still resident in the catchment areas of the cancer registries, and a proportion of these subjects will have migrated out of these areas. To allow for this migration, the person-years at risk were reduced by adjustment factors, specific for strata of year of birth, calendar period and city, which were estimated from data in the Adult Health Study subcohort of the Life Span Study (Sposto and Preston, 1992; Thompson et al, 1994).

### Statistical analysis

Person-years at risk were calculated using the *Person Years* computer program (Coleman et al, 1989). Relative risks were calculated by Poisson regression using the GLIM-4 statistical system (Francis et al, 1993). All analyses were stratified by attained age (< 40, 40–44, 45–49, 50–54, 55–59, 60–64, 65–69, 70–74, 75–79, 80+); calendar period (1969–1974, 1975–1979, 1980–1984, 1985–1989, 1990–1994); city of residence at the time of the bombing (Hiroshima, Nagasaki); age at the time of the bombing (< 15, 15+); and breast dose in Sv (0, 0.01–0.06, 0.07–0.30, 0.31+, missing). Cut points for the stratification variables were based on previous findings (Thompson et al, 1994; Goodman et al, 1997).

For those women who returned questionnaires in both Survey 1 and Survey 2 ($n$ = 11 067), person-years at risk were calculated in relation to diet as reported in Survey 1 until the date of entry to Survey 2, after which person-years were calculated in relation to diet as reported in Survey 2. To explore the possibility that a more reliable indication of usual long-term diet might be obtained by using the information from both Survey 1 and Survey 2 for follow-up after Survey 2, a subanalysis was conducted in which dietary data from the two surveys were combined.

### RESULTS

### Stratification variables

Table 1 shows the associations of attained age, calendar period, city, age at time of bombing and radiation dose with breast cancer risk, with these factors adjusted for each other. The risk of breast cancer was highest at ages 55–59. Risk increased significantly with calendar period, with rates in 1990–1994 over twice as high as those in 1969–1974; this increase in risk with calendar period

© 1999 Cancer Research Campaign

**Table 1** Relative risk of breast cancer (& 95% CI) for the baseline factors, adjusted for each other

| Factor | No. cases | Person-years[a] | Relative risk (95% CI) | Trend[b] |
|---|---|---|---|---|
| Attained age | | | | |
| <40 | 18 | 38 987 | 0.85 (0.43–1.69) | |
| 40–44 | 32 | 37 448 | 1.27 (0.74–2.18) | |
| 45–49 | 46 | 51 239 | 1.19 (0.75–1.88) | |
| 50–54 | 54 | 56 695 | 1.21 (0.80–1.83) | |
| 55–59 | 71 | 61 619 | 1.34 (0.94–1.92) | |
| 60–64 | 59 | 64 447 | 1.00[c] | |
| 65–69 | 48 | 55 024 | 0.98 (0.67–1.44) | |
| 70–74 | 39 | 45 768 | 0.98 (0.65–1.48) | |
| 75–79 | 27 | 36 736 | 0.83 (0.52–1.31) | |
| ≥80 | 33 | 41 026 | 0.86 (0.56–1.32) | $P = 0.086$ |
| Calendar period | | | | |
| 1969–1974 | 43 | 86 411 | 0.49 (0.34–0.73) | |
| 1975–1979 | 55 | 81 947 | 0.67 (0.48–0.94) | |
| 1980–1984 | 96 | 116 227 | 0.74 (0.57–0.97) | |
| 1985–1989 | 133 | 118 858 | 1.00[c] | |
| 1990–1994 | 100 | 85 546 | 1.10 (0.84–1.44) | $P < 0.0001$ |
| City | | | | |
| Hiroshima | 338 | 382 152 | 1.00[c] | |
| Nagasaki | 89 | 106 837 | 0.84 (0.67–1.07) | NA |
| Age at the bomb | | | | |
| <15 year | 132 | 144 317 | 0.92 (0.66–1.30) | |
| ≥15 years | 295 | 344 672 | 1.00[c] | NA |
| Radiation dose | | | | |
| Zero | 161 | 234 528 | 1.00[c] | |
| 0.01–0.06 Sv | 92 | 129 146 | 1.03 (0.80–1.33) | |
| 0.07–0.30 Sv | 56 | 58 039 | 1.36 (1.00–1.84) | |
| ≥ 0.31 Sv | 73 | 37 379 | 2.63 (1.99–3.48) | $P < 0.0001$ |
| Unknown | 45 | 29 897 | 2.03 (1.45–2.85) | |

[a]Adjusted for the probability of not having migrated out of the area covered by the cancer registries. [b]Test for linear trend; NA=not applicable. [c]Reference group.

was only slightly reduced by further adjustments for age at menarche, parity, age at first birth and body mass index (results not shown). Risk was not significantly associated with city of residence or with age at time of bombing. Risk increased significantly with increasing radiation dose to the breast. All subsequent analyses were adjusted for the variables in Table 1 by stratification with the categories as given in the Table.

### Reproductive, anthropometric and other non-dietary factors

Table 2 shows the associations of reproductive, anthropometric and other non-dietary factors with breast cancer risk. Risk decreased significantly with increasing age at menarche and increased significantly with increasing age at menopause. Risk decreased significantly with increasing parity and was non-significantly higher in women with a late age at first birth than in those with an early age at first birth; a combination of these variables indicated that risk decreased with both increasing parity and with decreasing age at first birth.

Risk was not significantly associated with weight or height but increased significantly with increasing body mass index. The relationship of body mass index with breast cancer risk was confined to diagnoses at ages ≥50 years; relative risks in the highest category compared to the lowest category were 0.93 (95% confidence interval (CI) 0.44–1.97) and 1.45 (1.04–2.00) for diagnoses at ages < 50 and ≥50 years respectively.

Risk was not significantly associated with use of hormone compounds, smoking, or alcohol consumption. Risk was significantly higher in women who were more educated than in those who were less educated.

### Soya foods and other dietary factors

Table 3 shows the associations of breast cancer risk with tofu, miso soup and 17 other foods and drinks. In each case, the lowest level of consumption was used as the reference group.

Tofu and miso soup were not significantly associated with breast cancer risk; relative risks in the highest category of consumption were 1.07 (0.78–1.47) and 0.87 (0.68–1.12) respectively. Further adjustment for parity and age at first birth, age at menarche, age at menopause, body mass index and education level had little effect on these estimates (results not shown). The intakes of tofu and miso soup were positively correlated (data not shown), and the risk for breast cancer in women with a relatively high total intake of tofu and miso soup, compared to women with a relatively low total intake of these foods, was 0.94 (0.73–1.20).

The associations of soya foods with breast cancer risk were examined separately for diagnoses at ages < 50 and ages ≥ 50. At ages < 50, relative risks were 1.30 (0.82–2.08) and 1.16 (0.56–2.38) comparing the medium and highest intake categories for tofu to the lowest; the corresponding figures for the ≥ 50 group were 0.92 (0.72–1.17) and 1.05 (0.73–1.49). For miso soup, the relative risks were 1.16 (0.69–1.95) and 1.03 (0.61–1.72) among women diagnosed before age 50 and 1.00 (0.76–1.31) and 0.83 (0.63–1.10) for women diagnosed at older ages.

We also examined the association of soya foods with breast cancer rates in the subgroup of women who had been exposed to

 *© 1999 Cancer Research Campaign*

**Table 2** Relative risk of breast cancer (& 95% CI) for reproductive and other non-dietary factors, each adjusted for attained age, calendar period, city, age at time of bombing and radiation dose

| Factor | No. cases | Person-years[a] | Relative risk (95% CI) | Trend[b] |
|---|---|---|---|---|
| Age at menarche (years) | | | | |
| <14 | 102 | 93 969 | 1.48 (1.12–1.96) | |
| 14 | 99 | 106 509 | 1.29 (0.98–1.71) | |
| 15 | 88 | 101 151 | 1.22 (0.92–1.62) | |
| ≥16 | 111 | 155 737 | 1.00[c] | *P* = 0.005 |
| Unknown | 27 | 31 621 | 1.11 (0.73–1.70) | |
| Age at menopause (years) | | | | |
| <48 | 67 | 93 477 | 1.00[c] | |
| 48–49 | 38 | 50 372 | 1.04 (0.70–1.55) | |
| 50–51 | 57 | 67 534 | 1.18 (0.83–1.68) | |
| ≥52 | 53 | 54 296 | 1.38 (0.96–1.98) | *P* = 0.043 |
| Unknown or not applicable | 212 | 223 311 | 1.39 (1.02–1.90) | |
| Parity | | | | |
| Nulliparous | 67 | 50 009 | 1.93 (1.44–2.58) | |
| 1–2 full-term pregnancies | 174 | 189 745 | 1.26 (0.99–1.59) | |
| ≥3 full-term pregnancies | 142 | 207 858 | 1.00[c] | *P* < 0.0001 |
| unknown | 44 | 41 376 | 1.61 (1.14–2.25) | |
| Age at first birth (years) | | | | |
| <22 | 52 | 82 438 | 0.79 (0.56–1.10) | |
| 22–23 | 64 | 91 325 | 0.83 (0.60–1.13) | |
| 24–25 | 86 | 93 558 | 1.08 (0.81–1.43) | |
| ≥26 | 104 | 119 389 | 1.00[c] | *P* = 0.081 |
| Unknown or not applicable | 121 | 102 278 | 1.35 (1.04–1.76) | |
| Combined parity and age at first birth (number of full-term pregnancies/age at first birth) | | | | |
| Nulliparous | 67 | 50 284 | 2.06 (1.47–2.90) | |
| 1–2/≥24 | 108 | 115 593 | 1.37 (0.99–1.89) | |
| 1–2/<24 | 41 | 54 340 | 1.12 (0.75–1.67) | |
| ≥3/≥24 | 67 | 81 998 | 1.25 (0.89–1.76) | |
| ≥3/<24 | 67 | 108 309 | 1.00[c] | NA |
| Unknown | 77 | 78 465 | 1.51 (1.08–2.10) | |
| Weight (kg) | | | | |
| <45 | 71 | 98 398 | 1.00[c] | |
| 45–49 | 88 | 111 575 | 1.05 (0.77–1.44) | |
| 50–54 | 107 | 114 420 | 1.20 (0.88–1.62) | |
| ≥55 | 143 | 142 825 | 1.25 (0.93–1.67) | *P* = 0.068 |
| Unknown | 18 | 21 772 | 1.12 (0.67–1.88) | |
| Height (cm) | | | | |
| <150 | 109 | 129 169 | 1.00[c] | |
| 150–152 | 111 | 124 177 | 1.02 (0.78–1.33) | |
| 153–155 | 74 | 92 364 | 0.90 (0.67–1.22) | |
| ≥156 | 107 | 112 649 | 1.05 (0.80–1.39) | *P* = 0.861 |
| Unknown | 26 | 30 629 | 1.03 (0.67–1.58) | |
| Body mass index (kg $m^{-2}$) | | | | |
| <20 | 95 | 125 591 | 1.00[c] | |
| 20–22.4 | 118 | 141 640 | 1.05 (0.80–1.38) | |
| 22.5–24.9 | 93 | 105 593 | 1.07 (0.80–1.43) | |
| ≥25 | 86 | 76 649 | 1.37 (1.02–1.84) | *P* = 0.050 |
| Unknown | 35 | 39 517 | 1.20 (0.81–1.78) | |
| Used hormone compounds | | | | |
| No | 340 | 393 297 | 1.00[c] | |
| Yes | 42 | 48 813 | 0.95 (0.69–1.31) | NA |
| Unknown | 45 | 46 878 | 1.06 (0.78–1.44) | |
| Smoking | | | | |
| Never smoked | 337 | 384 123 | 1.00[c] | |
| Past or current smoker | 68 | 70 431 | 1.05 (0.81–1.36) | NA |
| Unknown | 22 | 34 434 | 0.84 (0.55–1.29) | |
| Alcohol drinker | | | | |
| No | 315 | 368 282 | 1.00[c] | |
| Yes | 75 | 81 723 | 0.96 (0.74–1.23) | NA |
| Unknown | 37 | 38 985 | 1.14 (0.81–1.60) | |
| Education level | | | | |
| None or elementary | 152 | 203 775 | 0.80 (0.65–0.99) | |
| Secondary and above | 257 | 260 725 | 1.00[c] | NA |
| Unknown | 18 | 24 489 | 0.79 (0.49–1.27) | |

[a]Adjusted for the probability of not having migrated out of the area covered by the cancer registries. [b]Test for linear trend; NA = not applicable. [c]Reference group.

© 1999 Cancer Research Campaign

**Table 3** Relative risk of breast cancer (& 95% CI) by level of consumption for various foods, each adjusted for attained age, calendar period, city, age at time of bombing and radiation dose

| Food | No. cases | Person-years[a] | Relative risk (95% CI) | Trend[b] |
|---|---|---|---|---|
| *Soya foods* | | | | |
| Tofu | | | | |
| ≤1/week | 139 | 164 476 | 1.00[c] | |
| 2–4/week | 199 | 219 025 | 0.99 (0.80–1.24) | |
| ≥5/week | 52 | 52 695 | 1.07 (0.78–1.47) | *P* = 0.712 |
| Unknown | 37 | 52 794 | 0.91 (0.63–1.30) | |
| Miso soup | | | | |
| ≤1/week | 134 | 157 190 | 1.00[c] | |
| 2–4/week | 130 | 132 875 | 1.03 (0.81–1.31) | |
| ≥5/week | 123 | 156 123 | 0.87 (0.68–1.12) | *P* = 0.306 |
| Unknown | 40 | 42 799 | 1.13 (0.79–1.60) | |
| *Other foods and drinks* | | | | |
| Ham/sausage | | | | |
| ≤1/week | 218 | 224 931 | 1.00[c] | |
| 2–4/week | 100 | 121 092 | 0.88 (0.69–1.12) | |
| ≥5/week | 17 | 23 901 | 0.78 (0.48–1.28) | *P* = 0.137 |
| Unknown | 92 | 119 064 | 0.92 (0.71–1.18) | |
| Other meat | | | | |
| ≤1/week | 90 | 123 575 | 1.00[c] | |
| 2–4/week | 164 | 179 442 | 1.15 (0.89–1.50) | |
| ≥5/week | 119 | 115 815 | 1.12 (0.85–1.49) | *P* = 0.469 |
| Unknown | 54 | 70 157 | 0.99 (0.70–1.38) | |
| Fish (not dried) | | | | |
| ≤1/week | 99 | 125 089 | 1.00[c] | |
| 2–4/week | 159 | 185 031 | 1.08 (0.84–1.39) | |
| ≥5/week | 118 | 112 564 | 1.17 (0.90–1.54) | *P* = 0.209 |
| Unknown | 51 | 66 305 | 0.92 (0.66–1.29) | |
| Dried fish | | | | |
| ≤1/week | 259 | 256 264 | 1.00[c] | |
| 2–4/week | 64 | 81 898 | 0.85 (0.64–1.12) | |
| ≥5/week | 7 | 16 264 | 0.49 (0.24–1.02) | *P* = 0.029 |
| Unknown | 97 | 134 563 | 0.77 (0.60–0.98) | |
| Milk | | | | |
| ≤1/week | 150 | 167 029 | 1.00[c] | |
| 2–4/week | 85 | 84 636 | 1.06 (0.81–1.39) | |
| ≥5/week | 121 | 134 904 | 0.96 (0.76–1.22) | *P* = 0.770 |
| Unknown | 71 | 102 420 | 0.87 (0.66–1.16) | |
| Butter/cheese | | | | |
| ≤1/week | 179 | 212 831 | 1.00[c] | |
| 2–4/week | 79 | 76 878 | 1.27 (0.97–1.66) | |
| ≥5/week | 62 | 70 193 | 1.13 (0.85–1.51) | *P* = 0.239 |
| Unknown | 107 | 129 088 | 1.13 (0.89–1.45) | |
| Eggs | | | | |
| ≤1/week | 87 | 115 648 | 1.00[c] | |
| 2–4/week | 187 | 177 703 | 1.28 (0.99–1.66) | |
| ≥5/week | 126 | 155 444 | 1.05 (0.79–1.38) | *P* = 0.936 |
| Unknown | 27 | 40 195 | 0.95 (0.61–1.46) | |
| Rice | | | | |
| ≤1/day | 30 | 38 435 | 1.00[c] | |
| 2/day | 225 | 237 530 | 1.24 (0.85–1.81) | |
| ≥3/day | 153 | 191 762 | 1.13 (0.76–1.68) | *P* = 0.901 |
| Unknown | 19 | 21 262 | 1.27 (0.71–2.27) | |
| Bread | | | | |
| ≤1/day | 309 | 334 348 | 1.00[c] | |
| 2/day | 15 | 15 976 | 1.01 (0.60–1.70) | |
| ≥3/day | 2 | 1 411 | 1.66 (0.44–6.16) | *P* = 0.709 |
| Unknown | 101 | 137 253 | 0.84 (0.67–1.06) | |
| Western-style confectionery | | | | |
| ≤1/week | 147 | 169 788 | 1.00[c] | |
| 2–4/week | 127 | 156 235 | 0.90 (0.71–1.14) | |
| ≥5/week | 78 | 87 696 | 0.90 (0.68–1.18) | *P* = 0.403 |
| Unknown | 75 | 75 270 | 1.22 (0.92–1.62) | |
| Fruit | | | | |
| ≤1/week | 59 | 68 608 | 1.00[c] | |
| 2–4/week | 121 | 125 626 | 1.07 (0.78–1.46) | |
| ≥5/week | 222 | 258 661 | 0.95 (0.71–1.27) | *P* = 0.531 |
| Unknown | 25 | 36 093 | 0.87 (0.55–1.39) | |

 *© 1999 Cancer Research Campaign*

**Table 3** Continued

| Food | No. cases | Person-years[a] | Relative risk(95% CI) | Trend[b] |
|---|---|---|---|---|
| Green/yellow vegetables[d] | | | | |
| ≤1/week | 50 | 46 428 | 1.00[c] | |
| 2–4/week | 118 | 103 241 | 1.06 (0.76–1.48) | |
| ≥5/week | 67 | 63 650 | 0.99 (0.69–1.44) | *P* = 0.949 |
| Unknown | 192 | 275 669 | 1.03 (0.72–1.46) | |
| Sea vegetables | | | | |
| ≤1/week | 128 | 138 711 | 1.00[c] | |
| 2–4/week | 162 | 182 098 | 0.88 (0.70–1.11) | |
| ≥5/week | 106 | 115 651 | 0.89 (0.69–1.16) | *P* = 0.417 |
| Unknown | 31 | 52 529 | 0.68 (0.46–1.00) | |
| Pickled vegetables[e] | | | | |
| ≤1/week | 74 | 106 305 | 1.00[c] | |
| 2–4/week | 93 | 85 351 | 1.50 (1.10–2.03) | |
| ≥5/week | 224 | 238 697 | 1.35 (1.04–1.75) | *P* = 0.059 |
| Unknown | 36 | 58 638 | 0.98 (0.66–1.46) | |
| Coffee | | | | |
| ≤1/week | 151 | 184 263 | 1.00[c] | |
| 2–4/week | 71 | 84 676 | 1.03 (0.78–1.37) | |
| ≥5/week | 122 | 113 745 | 1.19 (0.93–1.52) | *P* = 0.258 |
| Unknown | 83 | 106 306 | 1.11 (0.84–1.46) | |
| Black tea | | | | |
| ≤1/week | 223 | 223 411 | 1.00[c] | |
| 2–4/week | 64 | 84 014 | 0.85 (0.64–1.12) | |
| ≥5/week | 55 | 54 009 | 1.10 (0.82–1.48) | *P* = 0.981 |
| Unknown | 85 | 127 555 | 0.76 (0.59–0.98) | |
| Green tea | | | | |
| ≤1/day | 54 | 57 846 | 1.00[c] | |
| 2–4/day | 251 | 270 447 | 1.02 (0.76–1.36) | |
| ≥5/day | 100 | 127 217 | 0.86 (0.62–1.21) | *P* = 0.284 |
| Unknown | 22 | 33 478 | 0.78 (0.47–1.29) | |

[a]Adjusted for the probability of not having migrated out of the area covered by the cancer registries. [b]Test for linear trend. [c]Reference level. [d]Recorded in Survey 2 only. [e]Including salted fish gut.

**Table 4** Relative risk of breast cancer (& 95% CI) among women in both surveys by level of consumption for soya foods, each adjusted for attained age, calendar period, city, age at time of bombing and radiation dose

| Food | No. cases | Person-years[a] | Relative risk (95% CI) | Trend[b] |
|---|---|---|---|---|
| Tofu[c] | | | | |
| Low | 35 | 42 071 | 1.00[d] | |
| Medium | 33 | 30 506 | 1.30 (0.81–2.09) | |
| High | 12 | 12 094 | 1.19 (0.62–2.29) | *P* = 0.436 |
| Unknown | 14 | 18 674 | 0.92 (0.49–1.71) | |
| Miso soup[c] | | | | |
| Low | 32 | 30 458 | 1.00[d] | |
| Medium | 29 | 27 204 | 1.05 (0.63–1.74) | |
| High | 23 | 30 771 | 0.74 (0.43–1.28) | *P* = 0.315 |
| Unknown | 10 | 14 913 | 0.68 (0.33–1.39) | |

[a]Adjusted for the probability of not having migrated out of the area covered by the cancer registries. [b]Test for linear trend. [c]Low = ≤1/week in both surveys, or one ≤1/week and the other 2–4/week; Medium = 2–4/week in both surveys, or one ≤1/week and the other ≥5/week; High = ≥5/week in both surveys, or one 2–4/week and the other ≥5/week. [d]Reference level.

very little radiation at the time of the atomic bombings (zero or 0.01–0.06 mSv); the results were similar to those for all women (results not shown).

Of the other foods and drinks examined, the only significant associations were a decrease in risk with increasing consumption of dried fish and an increase in risk with medium and high consumption of pickled vegetables. Further adjustment of the relative risks associated with tofu and miso soup for the consumption of both dried fish and pickled vegetables changed the relative risks for consumption five or more times per week to 1.07 (0.78–1.48) for tofu and 0.80 (0.61–1.03) for miso soup.

### Subanalysis of soya food consumption among women with dietary information in both Survey 1 and Survey 2

Of the 34 759 women studied, 11 067 had completed dietary questionnaires at both Survey 1 and Survey 2. The proportions of women reporting the same frequency of consumption in both surveys were 47.2% for tofu and 44.0% for miso soup. The proportions of women reporting extremely different consumption in the two surveys (i.e. once or less per week changed to five or more times per week, or vice versa) were 7.0% for tofu and 18.6% for miso soup.

© 1999 Cancer Research Campaign 

**Table 5** Epidemiological studies of soya consumption and breast cancer risk

| Study | Location | Cases | Menopausal status | Soya intake | Cases/ controls | Relative risks |
|---|---|---|---|---|---|---|
| *Case–control studies* | | | | | | |
| Hirohata et al, 1985 | Fukuoka, Japan | 212 | Combined | Cases mean 26 g of fat from soybean products/week$^{-1}$ | – | – |
| | | | | Controls mean 26 g of fat from soyabean products week$^{-1}$ | – | – |
| Lee et al, 1991, 1992 | Singapore | 109 | Premenopausal | <20.3 g products day$^{-1}$ | 41/53 | 1.0 (ref.) |
| | | | | 20.3–54.9 g products day$^{-1}$ | 38/73 | 0.6 (0.3–1.2) |
| | | | | 55.0+ g products/$^{d-1}$ | 30/81 | 0.4 (0.2–0.9) |
| | | 91 | Post-menopausal | <20.3 g products day$^{-1}$ | 37/87 | 1.0 (ref.) |
| | | | | 20.3–54.9 g products day$^{-1}$ | 26/66 | 0.9 (0.4–1.9) |
| | | | | 55.0+ g products day$^{-1}$ | 28/60 | 1.1 (0.5–2.3) |
| Hirose et al, 1995 | Aichi, Japan | 607 | Premenopausal | Bean curd ≤3 months$^{-1}$ | 86/2151 | 1.0 (ref.) |
| | | | | Bean curd 1–2 week$^{-1}$ | 270/6291 | 0.93 (0.72–1.19) |
| | | | | Bean curd ≥3 week$^{-1}$ | 250/6433 | 0.78 (0.60–1.00) |
| | | | | Miso <daily | 383/8615 | 1.0 (ref.) |
| | | | | Miso daily | 224/6268 | 1.16 (0.98–1.37) |
| | | 445 | Post-menopausal | Bean curd ≤3 months$^{-1}$ | 50/657 | 1.0 (ref.) |
| | | | | Bean curd 1–2 week$^{-1}$ | 151/2245 | 0.89 (0.64–1.24) |
| | | | | Bean curd ≥3 week$^{-1}$ | 242/3284 | 0.96 (0.70–1.31) |
| | | | | Miso <daily | 276/3901 | 1.0 (ref.) |
| | | | | Miso daily | 167/2291 | 0.96 (0.78–1.17) |
| Yuan et al, 1995 | Shanghai | 534 | Combined[a] | Per 18 g protein day$^{-1}$ Median controls 3.5 g day$^{-1}$ | – | 0.9 (0.6–1.3) |
| | Tianjin | 300 | Combined[a] | Per 18 g protein day$^{-1}$ Median controls 2.8 g day$^{-1}$ | – | 1.4 (0.7–3.0) |
| | Both centres | 834 | Combined[a] | Per 18 g protein day$^{-1}$ | – | 1.0 (0.7–1.4) |
| Wu et al, 1996 | US | 597 | Combined[a] | Tofu ≤12 year$^{-1}$ | 209/289 | 1.0 (ref.) |
| | | | | Tofu 13–42 year$^{-1}$ | 135/199 | 0.97 |
| | | | | Tofu 43–54 year$^{-1}$ | 138/232 | 0.84 |
| | | | | Tofu 55+ year$^{-1}$ | 114/238 | 0.67 |
| Witte et al, 1997 | US and Canada | 140 | Premenopausal | Tofu <1 week$^{-1}$ | not given | 1.0 (ref.) |
| | | | | Tofu 1 week$^{-1}$ | not given | 0.5 (0.2–1.1) |
| *Prospective studies* | | | | | | |
| Hirayama 1990 | Japan | 241 | Age 40+ | Miso soup <1 day$^{-1}$ | not given | 1.0 (ref.) |
| | | | | Miso soup ≥1 day$^{-1}$ | not given | 0.85 (0.68–1.06) |
| Greenstein et al 1996 | US | 1018 | Postmenopausal | Soya or tofu never | not given | 1.0 (ref.) |
| | | | | Soya or tofu consumers | not given | 0.76 (0.50–1.18) |
| *Study of husbands*[b] | | | | | | |
| Nomura et al 1978 | Hawaii | 86 | Combined | Food | Mean intake | |
| | | | | Tofu, cases | 117.1 g/week$^{-1}$ | |
| | | | | Tofu, controls | 150.8 g/week$^{-1}$ | |
| | | | | Miso soup, cases | 170.9 g/week$^{-1}$ | |
| | | | | Miso soup, controls | 279.5 g week$^{-1}$ | |

[a]Results did not differ when premenopausal and postmenopausal women were analysed separately. [b]Values in cases are values reported by husbands of women with breast cancer, most of whom were diagnosed before husband was interviewed. Results are from 1971–1975 survey.

*© 1999 Cancer Research Campaign*

To utilize these two measurements, we examined the relative risk for breast cancer in these women during the follow-up period after Survey 2, combining the data from both questionnaires (Table 4). Consumption of tofu and miso soup remained unassociated with breast cancer risk: relative risks in the highest category of consumption were 1.19 (0.62–2.29) and 0.74 (0.43–1.28) respectively.

### DISCUSSION

This study showed no significant associations between reported consumption of tofu or miso soup and breast cancer risk. Relatively high consumption of tofu was associated with a small increase in risk whereas relatively high consumption of miso soup was associated with a small decrease in risk. Adjusting for other risk factors for breast cancer did not materially alter these results. The reduction in risk associated with frequent consumption of miso soup was greater after further adjustment for consumption of dried fish and pickles but was still only of borderline statistical significance.

The strengths of this study are that the number of cases was relatively large, the dietary data were collected prospectively, and there was a wide range in the reported intakes of soya foods. The weaknesses of this study are that the dietary questionnaire did not include portion sizes, did not include all major foods, and has not been validated, so that it is not possible to estimate the intake of soya isoflavones or nutrients. Misclassification of dietary exposures is likely to cause underestimation of any true associations of dietary components with risk. Also, some cases of breast cancer among emigrants from Hiroshima and Nagasaki may have been missed, but this would not bias the results unless migration was strongly associated with diet.

The findings of previous studies of soya foods and breast cancer risk are summarized in Table 5. The early case–control study of Hirohata et al (1985) reported identical mean consumption of fat from soyabean products in cases and population controls. Lee et al (1991, 1992) reported a reduction in breast cancer risk with high soya consumption among premenopausal women in Singapore, but no associations were observed among post-menopausal women. The larger case–control studies of Hirose et al (1995) and Yuan et al (1995), conducted in populations with high soya consumption in Japan and China, respectively, found little evidence for an association of soya consumption with breast cancer risk; there was one significant association, a reduction in risk with high tofu consumption in premenopausal women in the Japanese study. Wu et al (1996) reported a reduction in breast cancer risk with increasing tofu consumption among Asian-Americans, but tofu consumption was much less frequent in this population than in the studies conducted in Asia, with a top frequency of only 55+ times per year. A nonsignificant reduction in risk in association with consumption of tofu at least once a week was also reported by Witte et al (1997) in a small study of North American women with bilateral breast cancer.

Only two previous prospective studies have reported on soya consumption and breast cancer risk. In a Japanese cohort, Hirayama (1990) reported a non-significant decrease in risk for daily consumption of miso soup relative to less than daily consumption. Greenstein et al (1996) reported a non-significant reduction in risk among soya consumers relative to non-consumers, but few women in this North American cohort consumed soya.

Two recent studies have reported that women with newly diagnosed breast cancer had lower urinary excretion of phyto-oestrogens than control women (Ingram et al, 1997; Zheng et al, 1999). However, these results could be due to an effect of illness or anxiety on diet and/or metabolism, and in both studies urinary nitrogen excretion was lower in cases than in controls, suggesting that the cases had a relatively low intake of protein and perhaps a low food intake at the time they were studied.

The current analysis of the Life Span Study showed, as in the previous analysis of Goodman et al (1997), that breast cancer risk was associated with the well established risk factors, including age, age at menarche, age at menopause, parity and age at first birth, and body mass index. The only statistically significant associations of breast cancer risk with dietary factors were a decrease in risk with increasing consumption of dried fish and an increase in risk with high consumption of pickled vegetables; these associations were not prior hypotheses and, because of the large number of comparisons made, they may be due to chance. The absence of significant associations of breast cancer risk with the other foods and drinks examined is compatible with the results of other epidemiological studies of nutrition and breast cancer (Hunter and Willett, 1996).

This cohort is unusual in that many women were exposed to high doses of ionizing radiation, and this is strongly related to breast cancer risk, as documented in previous studies in this cohort (e.g. Tokunaga et al, 1994). In contrast to previous reports on breast cancer incidence in the Life Span Study we did not observe a higher risk for women who were very young at the time of the bombing. This is because the highest risk associated with exposure to radiation in previous reports was among women who were under age 20 at the time of the bombing and diagnosed before age 35; all these cases were diagnosed before 1979 (Tokunaga et al, 1994), and few of them contribute to the current analysis. In this investigation, radiation exposure was only of interest as a potential confounder or effect modifier of any association of soya consumption with breast cancer risk. All the analyses of dietary factors and cancer risk were adjusted for radiation exposure; furthermore, the results were similar in the subgroup of women exposed to very little radiation. The clear associations of breast cancer risk with established hormonally mediated risk factors suggest that radiation does not mask hormonally mediated effects, and both Land et al (1994*a*, 1994*b*) and Goodman et al (1997) concluded that reproductive factors affect the risk for breast cancer in this cohort irrespective of the exposure to radiation. We therefore think that it is unlikely that the absence of an association between soya consumption and breast cancer risk is due to the substantial role of radiation as a cause of breast cancer in this cohort.

Another unusual characteristic of this cohort is the strong relationship of breast cancer risk with calendar period. The increase in breast cancer rates between the early 1970s and the early 1990s has been described before both in data from the Hiroshima and Nagasaki cancer registries (Goodman et al, 1994) and in Japan as a whole (Watanabe, 1993; Nagata et al, 1997*b*). The cause of this increase is not known. Nagata et al (1997*b*) estimated that under 40% of the increase between 1959–1960 and 1983–1987 could be accounted for by changes in age at menarche, age at first birth, parity and age at menopause, suggesting that other powerful risk factors must exist. Similarly, in the current analysis, we found that further adjustments for the well established breast cancer risk factors did not substantially reduce the increase in breast cancer risk with calendar period. Changes in diet may be involved in this

© 1999 Cancer Research Campaign increase. No clear associations with dietary factors were observed in the current analysis, but the questionnaire covered only a limited number of foods. It remains possible that dietary factors that could not be assessed, for example, total intakes of energy, fat and fibre, could be associated with breast cancer risk in this cohort.

### ACKNOWLEDGEMENTS

This publication is based on research performed at the Radiation Effects Research Foundation (RERF), Hiroshima and Nagasaki, Japan. RERF is a private nonprofit foundation funded equally by the Japanese Ministry of Health and Welfare and the US Department of Energy through the National Academy of Sciences. The authors thank the women who participated in this study; Eric Grant, Yukiko Hayashi, and Hiroko Moriwaki for data management and analyses; and Dr Dale Preston for assisting with the preparation of the data and commenting on the manuscript. TJ Key, PN Appleby, and V Beral were supported by the Imperial Cancer Research Fund, UK.

### REFERENCES

Cassidy A, Bingham S and Setchell KDR (1994) Biological effects of a diet of soy protein rich in isoflavones on the menstrual cycle of premenopausal women. *Am J Clin Nutr* **60**: 333–340

Cassidy A, Bingham S and Setchell K (1995) Biological effects of isoflavones in young women: importance of the chemical composition of soyabean products. *Br J Nutr* **74**: 587–601

Coleman MP, Hermon C and Douglas A (1989) Person-years (PYRS). A Fortran program for cohort study analysis. IARC Technical Report No. 89. Lyon: IARC

Collaborative Group on Hormonal Factors in Breast Cancer (1997) Breast cancer and hormone replacement therapy: collaborative reanalysis of data from 51 epidemiological studies of 52 705 women with breast cancer and 108 411 women without breast cancer. *Lancet* **350**: 1047–1059

Francis B, Green M and Payne C (eds) (1993) *The GLIM System Release 4 Manual*. Oxford University Press: Oxford

Goodman MT, Mabuchi K, Morita M, Soda M, Ochikubo S, Fukuhara T, Ikeda T and Terasaki M (1994) Cancer incidence in Hiroshima and Nagasaki, Japan, 1958–1987. *Eur J Cancer* **30A**: 801–807

Goodman MT, Cologne JB, Moriwaki H, Vaeth M and Mabuchi K (1997) Risk factors for primary breast cancer in Japan: 8-year follow-up of atomic bomb survivors. *Prev Med* **26**: 144–153

Greenstein J, Kushi L, Zheng W, Fee R, Campbell D, Sellers T and Folsom A (1996) Risk of breast cancer associated with intake of specific foods and food groups. *Am J Epidemiol* **143**: S36

Hirayama T (1990) Life-style and mortality: a large-scale census-based cohort study in Japan. In: *Contributions to Epidemiology and Biostatistics, Vol. 6* Wahrendorf J (ed) Karger: Basel

Hirohata T, Shigematsu T, Nomura AMY, Nomura Y, Horie A and Hirohata I (1985) Occurrence of breast cancer in relation to diet and reproductive history: a case-control study in Fukuoka, Japan. *Natl Cancer Institute Monogr* **69**: 187–190

Hirose K, Tajima K, Hamajima N, Inoue M, Takezaki T, Kuroishi T, Yoshida M and Tokudome S (1995) A large-scale, hospital-based case-control study of risk factors of breast cancer according to menopausal status. *Jpn J Cancer Res* **86**: 146–154

Hunter DJ and Willett WC (1996) Nutrition and breast cancer. *Cancer Causes Control* **7**: 56–68

Ingram D, Sanders K, Kolybaba M and Lopez D (1997) Case-control study of phyto-oestrogens and breast cancer. *Lancet* **350**: 990–994

Land CE, Hayakawa N, Machado SG, Yamada Y, Pike MC, Akiba S and Tokunaga M (1994*a*) A case-control interview study of breast cancer among Japanese A-bomb survivors. I. Main effects. Cancer Causes Control **5**: 157–165

Land CE, Hayakawa N, Machado SG, Yamada Y, Pike MC, Akiba S and Tokunaga M (1994*b*) A case-control interview study of breast cancer among Japanese A-bomb survivors. II. Interactions with radiation dose. Cancer Causes Control **5**: 167–176

Lee HP, Gourley L, Duffy SW, Estève J, Lee J and Day NE (1991) Dietary effects on breast-cancer risk in Singapore. *Lancet* **337**: 1197–1200

Lee HP, Gourley L, Duffy SW, Estève J, Lee J and Day NE (1992) Risk factors for breast cancer by age and menopausal status: a case-control study in Singapore. *Cancer Causes Control* **3**: 313–322

Lu L-JW, Anderson KE, Grady JJ and Nagamani M (1996) Effects of soya consumption for one month on steroid hormones in premenopausal women: implications for breast cancer risk reduction. *Cancer Epidemiol Biomarkers Prev* **5**: 63–70

Mabuchi K, Soda M, Ron E, Tokunaga M, Ochikubo S, Sugimoto S, Ikeda T, Terasaki M, Preston DL and Thompson DE (1994) Cancer incidence in atomic bomb survivors. Part I: Use of the tumor registries in Hiroshima and Nagasaki for incidence studies. *Radiat Res* **137**: S1–S16

Nagata C, Kabuto M, Kurisu Y and Shimizu H (1997*a*) Decreased serum estradiol concentration associated with high dietary intake of soy products in premenopausal Japanese women. *Nutr Cancer* **29**: 228–233

Nagata C, Kawakami N and Shimizu H (1997*b*) Trends in the incidence rate and risk factors for breast cancer in Japan. *Breast Cancer Res Treat* **44**: 75–82

Nomura A, Henderson BE and Lee J (1978) Breast cancer and diet among the Japanese in Hawaii. *Am J Clin Nutr* **31**: 2020–2025

Pike MC, Spicer DV, Dahmoush L and Press MF (1993) Estrogens, progestogens, normal breast cell proliferation, and breast cancer risk. *Epidemiol Rev* **15**: 17–35

Preston DL, Kato H, Kopecky K and Fujita S (1987) Studies of the mortality of A-bomb survivors. 8. Cancer mortality, 1950–1982. *Radiat Res* **111**: 151–178

Roesch WC (1987) Ed., *U.S.–Japan Joint Reassessment of Atomic Bomb Radiation Dosimetry in Hiroshima and Nagasaki*, Final Report, Vol. 1. Radiation Effects Research Foundation, Hiroshima

Setchell KDR, Borriello SP, Hulme P, Kirk DN and Axelson M (1984) Nonsteroidal estrogens of dietary origin: possible roles in hormone-dependent disease. *Am J Clin Nutr* **40**: 569–578

Sposto R and Preston DL (1992) Correcting for catchment area nonresidency in studies based on tumor-registry data. Technical report CR1-92, Radiation Effects Research Foundation, Hiroshima, Japan

Thomas HV, Reeves GK and Key TJA (1997) Endogenous estrogen and postmenopausal breast cancer: a quantitative review. *Cancer Causes Control* **8**: 922–928

Thompson D, Mabuchi K, Ron E, Soda M, Tokunaga M, Ochikubo S, Sugimoto S, Ikeda T, Terasaki M, Izumi S and Preston D (1994) Cancer incidence in atomic bomb survivors. Part II. Solid tumors, 1958–87. *Radiat Res* **137**: S17–S67

Tokunaga M, Land CE, Tokuoka S, Nishimori I, Soda M and Akiba S (1994) Incidence of female breast cancer among atomic bomb survivors, 1950–1985. *Radiat Res* **138**: 209–223

Watanabe S (1993) Breast cancer in Japan. Trends and recent researches in biology and epidemiology. *Asian Med J* **36**: 486–494

Witte JS, Ursin G, Siemiatycki J, Thompson WD, Paganini-Hill A and Haile RW (1997) Diet and premenopausal bilateral breast cancer: a case-control study. *Breast Cancer Res Treat* **42**: 243–251

Wu AH, Ziegler RG, Horn-Ross PL, Nomura AMY, West DW, Kolonel LN, Rosenthal JF, Hoover RN and Pike MC (1996) Tofu and risk of breast cancer in Asian-Americans. *Cancer Epidemiol Biomarkers Prev* **5**: 901–906

Yuan J-M, Wang Q-S, Ross RK, Henderson BE and Yu MC (1995) Diet and breast cancer in Shanghai and Tianjin, China. *Br J Cancer* **71**: 1353–1358

Zheng W, Dai Q, Custer LJ, Shu X-O, Wen W-Q, Jin F and Franke AA (1999) Urinary excretion of isoflavonoids and the risk of breast cancer. *Cancer Epidemiol Biomarkers Prev* **8**: 35–40

 *© 1999 Cancer Research Campaign*

## References

[PDF_00768] Cassidy A., Bingham S., Setchell K. D. (1994). Biological effects of a diet of soy protein rich in isoflavones on the menstrual cycle of premenopausal women.. Am J Clin Nutr.

[PDF_00771] Cassidy A., Bingham S., Setchell K. (1995). Biological effects of isoflavones in young women: importance of the chemical composition of soyabean products.. Br J Nutr.

[PDF_00785] Goodman M. T., Cologne J. B., Moriwaki H., Vaeth M., Mabuchi K. (1997). Risk factors for primary breast cancer in Japan: 8-year follow-up of atomic bomb survivors.. Prev Med.

[PDF_00782] Goodman M. T., Mabuchi K., Morita M., Soda M., Ochikubo S., Fukuhara T., Ikeda T., Terasaki M. (1994). Cancer incidence in Hiroshima and Nagasaki, Japan, 1958-1987.. Eur J Cancer.

[PDF_00794] Hirohata T., Shigematsu T., Nomura A. M., Nomura Y., Horie A., Hirohata I. (1985). Occurrence of breast cancer in relation to diet and reproductive history: a case-control study in Fukuoka, Japan.. Natl Cancer Inst Monogr.

[PDF_00797] Hirose K., Tajima K., Hamajima N., Inoue M., Takezaki T., Kuroishi T., Yoshida M., Tokudome S. (1995). A large-scale, hospital-based case-control study of risk factors of breast cancer according to menopausal status.. Jpn J Cancer Res.

[PDF_00801] Hunter D. J., Willett W. C. (1996). Nutrition and breast cancer.. Cancer Causes Control.

[PDF_00803] Ingram D., Sanders K., Kolybaba M., Lopez D. (1997). Case-control study of phyto-oestrogens and breast cancer.. Lancet.

[PDF_00805] Land C. E., Hayakawa N., Machado S. G., Yamada Y., Pike M. C., Akiba S., Tokunaga M. (1994). A case-control interview study of breast cancer among Japanese A-bomb survivors. I. Main effects.. Cancer Causes Control.

[PDF_00808] Land C. E., Hayakawa N., Machado S. G., Yamada Y., Pike M. C., Akiba S., Tokunaga M. (1994). A case-control interview study of breast cancer among Japanese A-bomb survivors. II. Interactions with radiation dose.. Cancer Causes Control.

[PDF_00814] Lee H. P., Gourley L., Duffy S. W., Estève J., Lee J., Day N. E. (1992). Risk factors for breast cancer by age and menopausal status: a case-control study in Singapore.. Cancer Causes Control.

[PDF_00812] Lee H. P., Gourley L., Duffy S. W., Estéve J., Lee J., Day N. E. (1991). Dietary effects on breast-cancer risk in Singapore.. Lancet.

[PDF_00817] Lu L. J., Anderson K. E., Grady J. J., Nagamani M. (1996). Effects of soya consumption for one month on steroid hormones in premenopausal women: implications for breast cancer risk reduction.. Cancer Epidemiol Biomarkers Prev.

[PDF_00821] Mabuchi K., Soda M., Ron E., Tokunaga M., Ochikubo S., Sugimoto S., Ikeda T., Terasaki M., Preston D. L., Thompson D. E. (1994). Cancer incidence in atomic bomb survivors. Part I: Use of the tumor registries in Hiroshima and Nagasaki for incidence studies.. Radiat Res.

[PDF_00825] Nagata C., Kabuto M., Kurisu Y., Shimizu H. (1997). Decreased serum estradiol concentration associated with high dietary intake of soy products in premenopausal Japanese women.. Nutr Cancer.

[PDF_00828] Nagata C., Kawakami N., Shimizu H. (1997). Trends in the incidence rate and risk factors for breast cancer in Japan.. Breast Cancer Res Treat.

[PDF_00830] Nomura A., Henderson B. E., Lee J. (1978). Breast cancer and diet among the Japanese in Hawaii.. Am J Clin Nutr.

[PDF_00832] Pike M. C., Spicer D. V., Dahmoush L., Press M. F. (1993). Estrogens, progestogens, normal breast cell proliferation, and breast cancer risk.. Epidemiol Rev.

[PDF_00835] Preston D. L., Kato H., Kopecky K., Fujita S. (1987). Studies of the mortality of A-bomb survivors. 8. Cancer mortality, 1950-1982.. Radiat Res.

[PDF_00840] Setchell K. D., Borriello S. P., Hulme P., Kirk D. N., Axelson M. (1984). Nonsteroidal estrogens of dietary origin: possible roles in hormone-dependent disease.. Am J Clin Nutr.

[PDF_00846] Thomas H. V., Reeves G. K., Key T. J. (1997). Endogenous estrogen and postmenopausal breast cancer: a quantitative review.. Cancer Causes Control.

[PDF_00849] Thompson D. E., Mabuchi K., Ron E., Soda M., Tokunaga M., Ochikubo S., Sugimoto S., Ikeda T., Terasaki M., Izumi S. (1994). Cancer incidence in atomic bomb survivors. Part II: Solid tumors, 1958-1987.. Radiat Res.

[PDF_00852] Tokunaga M., Land C. E., Tokuoka S., Nishimori I., Soda M., Akiba S. (1994). Incidence of female breast cancer among atomic bomb survivors, 1950-1985.. Radiat Res.

[PDF_00857] Witte J. S., Ursin G., Siemiatycki J., Thompson W. D., Paganini-Hill A., Haile R. W. (1997). Diet and premenopausal bilateral breast cancer: a case-control study.. Breast Cancer Res Treat.

[PDF_00860] Wu A. H., Ziegler R. G., Horn-Ross P. L., Nomura A. M., West D. W., Kolonel L. N., Rosenthal J. F., Hoover R. N., Pike M. C. (1996). Tofu and risk of breast cancer in Asian-Americans.. Cancer Epidemiol Biomarkers Prev.

[PDF_00863] Yuan J. M., Wang Q. S., Ross R. K., Henderson B. E., Yu M. C. (1995). Diet and breast cancer in Shanghai and Tianjin, China.. Br J Cancer.

[PDF_00865] Zheng W., Dai Q., Custer L. J., Shu X. O., Wen W. Q., Jin F., Franke A. A. (1999). Urinary excretion of isoflavonoids and the risk of breast cancer.. Cancer Epidemiol Biomarkers Prev.

